# Role of Ion Channels in the Chemotransduction and Mechanotransduction in Digestive Function and Feeding Behavior

**DOI:** 10.3390/ijms23169358

**Published:** 2022-08-19

**Authors:** Zhenya Zhu, Yuhao Wu, Ziyu Liu, Yuezhou Li, Mizu Jiang

**Affiliations:** 1 Endoscopy Center and Gastrointestinal Laboratory, Children’s Hospital, Zhejiang University School of Medicine, National Clinical Research Center for Child Health, National Children’s Regional Medical Center, Hangzhou 310052, China; 2 National Health Center and Chinese Academy of Medical Sciences Key Laboratory of Medical Neurobiology, Children’s Hospital, Zhejiang University School of Medicine, National Clinical Research Center for Child Health, National Children’s Regional Medical Center, Hangzhou 310052, China; 3 Department of Gastroenterology, Children’s Hospital, Zhejiang University School of Medicine, National Clinical Research Center for Child Health, National Children’s Regional Medical Center, Hangzhou 310052, China

**Keywords:** ion channels, mechanical stimulation, chemical stimulation, signaling transduction

## Abstract

The gastrointestinal tract constantly communicates with the environment, receiving and processing a wide range of information. The contents of the gastrointestinal tract and the gastrointestinal tract generate mechanical and chemical signals, which are essential for regulating digestive function and feeding behavior. There are many receptors here that sense intestinal contents, including nutrients, microbes, hormones, and small molecule compounds. In signal transduction, ion channels are indispensable as an essential component that can generate intracellular ionic changes or electrical signals. Ion channels generate electrical activity in numerous neurons and, more importantly, alter the action of non-neurons simply and effectively, and also affect satiety, molecular secretion, intestinal secretion, and motility through mechanisms of peripheral sensation, signaling, and altered cellular function. In this review, we focus on the identity of ion channels in chemosensing and mechanosensing in the gastrointestinal tract.

## 1. Introduction

The gastrointestinal tract (GIT) is rich in chemical [1] and physical [2] signals. The gastrointestinal tract is a tubular structure where food exerts pressure on the lumen, and specific receptors receive nutrients broken down by food in particular cells. Non-nutrients such as water, salt, inflammatory factors, neurotransmitters, and hormones also contribute to the digestion and absorption in the gut [3]. Gut microbiota has received increasing research attention in recent years because of its essential role in regulating intestinal homeostasis [4]. Gut microbes and their metabolites are widely distributed in the intestine, and various receptors can respond to commensal and harmful microbes [5]. Both nutrients and non-nutrients are indispensable sources of information in the GIT.

The gut is an important sensory organ [2,6] that integrates a large amount of information to modulate gastrointestinal function. Some cells in the intestine can sense chemical and mechanical stimuli. Previous studies have classified the intestinal epithelium components as intestinal stem cells, enterocytes, goblet cells, tuft cells, enteroendocrine cells (EECs), and Paneth cells [7]. Epithelial cells work together to create a semipermeable barrier and are responsible for digestion, absorption, and immunity [8,9,10]. EECs are responsible for chemical sensing [11], mucosal immunity [12], and brain-gut communication [13]. The intestinal epithelium is the component in direct contact with the intestinal contents, while the neurons in the intestine also have direct or indirect access to information about the intestinal contents. The gut receives signals from the central nervous system and also processes signals independently via the enteric nervous system (ENS) [14,15]. ENS consists of four neuronal subpopulations: motorneurons, sensory neurons, interneurons, and viscerofugal neurons. Each population plays different roles and contains a variety of biologically active substances, including ion channels. Neurons can sense mechanical and chemical stimuli with abundant receptors. Sensory neurons transmit signals to the intestine or brain. Smooth muscle cells (SMCs) are important in intestinal dynamics, responding to mechanical stimulation and electrical signals [16]. The intestinal epithelium, neurons, immune cells, and SMCs collaborate to perform sensory, motor, digestive, absorptive, and immune properties.

The components of cellular sensation machinery are multiple [1,2,6], such as the cytoskeleton, cell junctions, cellular matrix, G protein coupled receptor (GPCR), receptors, and ion channels. The function of mechanically and chemically sensitive ion channels is increasingly studied as a mediator capable of sensing external information. A process of information acquisition, encoding, transmission, and decoding is generated in the physiology of the GIT. Ion channels mediate ion flow and generate electrical signals or functional changes, and these effects occur in different spatial and temporal domains. There are some modalities in which ion channels are engaged in signal transduction: (1) peripheral sensing, where ion channels sense different stimuli to produce effects, such as perceiving mechanical forces [17] or specific chemical activators [18]; (2) electrical signaling, where membrane potential can be altered in the activated state of ion channels, such as in neurons where ion channels can produce depolarization or hyperpolarization by ion flow [11]; and (3) effector work, such as electrical signals and secretion coupling [19]. Transient receptor potential (TRP) [20] channels and Piezo [21] channels are two crucial types of channels in the GIT. TRP channels are capable of sensing temperature, pain, pressure, vision, and taste. Piezo channels are identified as mechanosensitive ion channels. In addition, other ion channels, such as acid-sensing ion channels (ASICs) in the GIT, sense mechanical and chemical stimuli.

## 2. Ion Channels Linking Stimuli to Molecular Secretion and Intestinal Secretion

**Figure 1:**
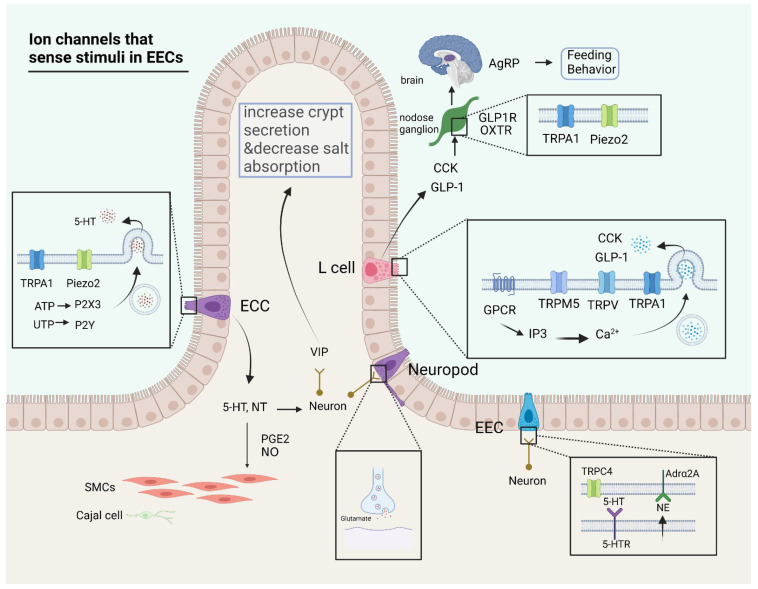
Ion channels that sense stimuli in enteroendocrine cells (EECs). The role of ion channels in signal sensation and transmission in EECs includes secretion of hormones, excitation of neurons, contraction of smooth muscle, and transmission of neuron-mediated signals. (1) 5-HT and neurotensin (NT) secreted by ECCs are related to calcium currents mediated by ion channels such as transient receptor potential ankyrin 1 (TRPA1) and Piezo2. Hormones such as 5-HT are able to elicit contractile activity in smooth muscle cells (SMCs), with the involvement of prostaglandin E2 (PGE2) and nitric oxide (NO). (2) L cells are capable of producing glucagon-like peptide-1 (GLP-1) and cholecystokinin (CCK) under the action of calcium currents, with the participation of G protein coupled receptor (GPCR), transient receptor potential melastatin 5 (TRPM5), transient receptor potential vanilloid (TRPV), TRPA1, which can generate satiety signals in the brain through the action of permutations in the nodal ganglia and thus control feeding behavior. (3) Neuropod cells are capable of producing synaptic connections directly with neurons and transmit signals for neurotransmitters through glutamate. (4) A portion of EECs are capable of producing bidirectional communication with neurons through norepinephrine (NE) and 5-HT as mediators. (created with BioRender.com).

EECs are considered a chemosensor and mechanosensor, with secreted hormones acting on proximal and distant tissues or organs [11,17]. EECs are previously classified according to the hormones they secrete. Such classifications include serotonin-secreting enterochromaffin cells (ECCs), histamine-secreting enterochromaffin-like cells, cholecystokinin (CCK)-secreting I cells, gastrin-secreting G cells, and glucagon-like peptide-1/2 (GLP-1/2)-secreting L cells. EECs are not only able to respond to nutrients through secretion but also communicate information to neurons through synapses. There are ion channels in EECs for signal sensing, decoding, and transforming electrical signals into physiological functions (Figure 1).

EECs receive mechanical and chemical stimuli through ion channels and regulate their secretory activities through complex signaling mechanisms. ECCs have electrical excitability owing to abundant Na channel and K channel expression [11], along with transient receptor potential ankyrin 1 (TRPA1) [11,18] for sensing chemical stimuli and Piezo2 [17,22] for sensing mechanical stimuli. Characterized by *ChgA* and *NeuroD1*, respectively, ECCs were activated by allyl isothiocyanate (AITC) and mechanical forces to generate inward Ca^2+^ currents. Ca^2+^ currents and vesicular cytosolic coupling result in the release of 5-HT with the involvement of adenosine triphosphate (ATP) -gated P2X3 receptors [23] and uridine triphosphate (UTP)-associated P2Y receptors [24]. Ion channels are able to participate in the secretory activity of ECCs as a direct current-generating component, which is generated after ion channel activation by stimulation and is coupled to multiple Ca signaling pathways. STC-1, a cellular model of EECs, can secrete CCK and GLP-1 via activation of TRPA1 [19,25,26] and transient receptor potential melastatin 5 (TRPM5) [27] in response to stimulation by specific activators, short-chain fatty acids, and vomitoxin. The process is accompanied by GPCR sensation of stimuli [28], Phospholipase (PLC) pathways mediating Ca^2+^ release, and Ca channels eliciting Ca^2+^ currents. Four associated and essential steps are required for EECs to sense external signals and generate secretory activity [28]: (1) the reception of stimulus signals, inward currents may be generated by chemosensitive or mechanosensitive ion channels, and GPCR may also be involved in this process; (2) the cell generates a depolarization potential, opening voltage-gated channels; (3) the elevation of intracellular Ca^2+^, due to the release of intracellular Ca^2+^ and the formation of inward currents by Ca channels; and (4) the release of hormone-containing vesicles triggered by elevated Ca^2+^. However, there are still many unknowns about the involvement of ion channels in the secretion of EECs. It is intriguing to note the mode of action of ion channels in hormone secretion at the cellular and tissue levels. Moreover, the role of ion channels in nutrient sensing has not been fully discovered. Transcriptome data to discriminate the identity of EECs revealed that many cell types may sense chemical and mechanical stimuli because of the expression of ion channels [29,30]. It requires further experiments to confirm the function of EECs expressing ion channels.

The role of EECs in neuro-epithelial crosstalk, in addition to hormone secretion, has been found to provide evidence for the formation of synaptic connections. A subset of EECs express α2A adrendoreceptor (Adrα2A), which is able to form a signaling cascade with transient receptor potential canonical 4 (TRPC4) to produce connections with adrenergic nerve fibers [11]. Furthermore, 5-HT, secreted by EECs, can activate the 5-HT receptor (5-HTR) in afferent nerves for signaling [11,31]. The vagus nerve is not in direct contact with the intestinal contents. However, it may have sensory afferents via EECs or other cells (tufted cells, immune cells, enteric neurons). The vagus nerve expresses receptors for intestinal hormones such as CCK, GLP-1, and Peptide YY (PYY) [32,33]. Transient receptor potential vanilloid (TRPV) channels (excluding TRPV1) have a role in CCK-induced depolarization of calcium current vagal membranes in afferent nerves [34]. The hormones secreted by EECs exert their effects locally and are closely related to neuronal activation and signaling. It is a slow process for the hormones secreted by EECs to reach their target organs for action, but there is a fast neuro-epithelial loop based on synaptic connections [13]. Neuropod cells form basal pseudopod-like processes [35] and are found to express pre- and postsynaptic proteins that form neural loops with the vagus nerve [36]. A further study [36,37] has shown that Neuropod cells use glutamate as a neurotransmitter that can rapidly process glucose stimuli. Neuropod cells are a new sensory transducer in the intestine that transmits information to the brain.

The ion channels involved in the intestine during absorption and secretion are described in the past [38]. Intestinal toxins trigger the secretion of 5-HT and neurotensin by EECs via cyclic adenosine monophosphate (cAMP) and cyclic guanosine monophosphate (cGMP), which affect neuronal activity in a paracrine manner, leading to the release of vasoactive intestinal peptide (VIP) to increase crypt secretion and decrease salt absorption [39]. Whether intestinal secretion is associated with mechanosensitive ion channels still needs further investigation. One study found that bile ducts cause elevated Ca via Piezo1 [40], which causes constriction of the bile ducts and secretion of bile. This suggests that mechanical factors may be an under-explored area of intestinal secretion. The main correlates of intestinal secretion and absorption are Na^+^, K^+^, Cl^−^, HCO_3_^−^, water, and solutes such as glucose [41]. The function of these ion channels is mainly related to voltage, ion concentration, and solute concentration. There is evidence that colonic mucosal TRPA1 is involved in regulating HCO_3_^−^ secretion via prostaglandin E2 (PGE2) [42].

A variety of hormones can perform regulatory physiological and pathological functions. LX1031, an inhibitor of tryptophan hydroxylase, a key enzyme for 5-HT synthesis, has been shown in clinical trials to be effective in treating diarrhea in non-constipated irritable bowel syndrome (IBS) [43]. GLP-1 acts on intestinal sympathetic neurons at the terminal ileum and regulates feeding by affecting gastric distension and the hypothalamus [44]. This suggests that the control of various hormones secreted by EECs may be an essential target for treating the disease. And control of the secretory activity of EECs can also be achieved through ion channels, a promising target.

## 3. Ion Channels Involved in the Induction of Nutrition Response and Satiety

Food intake is essential for maintaining homeostasis. The taste, aroma of food, and the energy influence feeding behavior [45,46]. Gastric satiety is mainly dependent on volumetric changes. Volumetric signals contribute to the regulation of feeding behavior in a negative feedback manner. On the other hand, the intestine is capable of nutritional sensation and regulates the body’s need for nutrients through neurohumoral regulation. Neuronal modulation is an essential aspect of feeding behavior regulation [47]. The vagus nerve plays a crucial role in mediating the production of satiety. When encoding the information it receives, the vagus nerve will integrate three parts of information: the organ, the tissue, and the stimulus pattern [48]. Ion channels sense chemical and mechanical stimuli, providing different stimulation patterns for the external nerve.

The GIT, as a hollow organ, inevitably receives pressure from the contents of the GIT, as well as forces from its motility and mutual compression. Mechanosensitive ion channels expressed in neurons act as primary mechanosensors, regulating food intake and nutritional responses. A specific knockout of *Piezo* and its homologs were found to increase feeding in *Drosophila* [49,50] and *C. elegans* [51], suggesting that Piezo is able to inhibit feeding through stress sensation. In addition, Piezo was also found to be expressed in the digestive tract, nerves, and brain, revealing a direct gut-brain neural loop that uses Piezo as a vital mediator of signal sensation to transmit signals for the digestive tract distension [49,50]. *Diuretic hormone 44 (DH44)* neurons sense nutrients and are stimulated by sugar. Piezo, activated by crop enlargement during feeding, inhibits the activity of *DH44* neurons and regulates feeding behavior [52]. Piezo-mediated feeding regulation depends on multiple neural loops, which inhibit *DH44* neuron activity and increase *Dlip2* neuron activity. Piezo channels may function as primary sensors of the filling stimulus.

In mammalian models, the Piezo2 intestinal vagal sensory neurons can directly sense stretch stimuli in the esophagus, stomach, and duodenum. This suggests that ion channels are instructive in determining the stimulation pattern of the vagus nerve [48]. The *GLP1R* and *OXTR* neurons of the vagus nerve constitute the mechanosensitive intraganglionic laminar endings (IGLEs) that can lead to reduced feeding [53,54], and these nerve fibers project to the nuclei of the solitary tract (NTS) area and inhibit hypothalamic hunger-promoting agouti-related peptide (AgRP) neurons. In addition, the intestine has ASICs responsible for mechanoreception, and the knockout of this channel affects visceral mechanotransduction [55]. The manipulation of gastrointestinal mechanosensation has shown reasonable control of feeding. Future studies on gastrointestinal mechanosensation and obesity will better exploit the auxiliary role of ion channels in human diseases.

The vagus nerve is an integral part of the brain-gut axis and bridges the gap between the intestine and the brain. Hormones and nutrients can serve as two factors that the vagus nerve perceives to mediate satiety. In *C. elegans*, ASIC-enteric serotonergic neurons (NSMs) act as sensory neurons that recognize ingestion and signal satiety to the brain; activation of NSMs during feeding also causes slow motility [56]. TRPA1 and TRPV1-positive neurons in the brain can sense CCK [57]. GLP-1 directly activates the paraventricular nucleus of the hypothalamus (PVH), a brain region that regulates feeding [58]. EECs secrete hormones in the intestine, and neurons can receive these signals to produce a reaction to nutrition. The ion channel mechanisms of neuronal chemosensing and hormone response in this process still need further exploration.

The vagus nerve is able to sense CCK, PYY, leptin, and GLP-1 secreted by EECs, forming a nutrition response [59]. This approach is indirect, with the vagus nerve performing the transmission of signals. Chemical signals can be regulated in the secretion of EECs, but there is no direct evidence that chemosensitive ion channels can transmit signals in satiety formation. However, many studies have shown that chemosensory ion channels can regulate the amount of food intake and even obesity. TRPA1 is also associated with the anorexic response, regulating the amount of food intake [60]. TRPV1 channels are associated with mechanosensory sensitivity of the gastric vagus nerve and mediate a decrease in sensitivity in obesity models [61]. The control of ion channels allows a direct adjustment of feeding. Lowering the threshold of mechanical stimulation and increasing the intensity of hormonal regulation both make it easier to develop a feeling of satiety. The development of relevant drugs helps to achieve the programming of feeding.

## 4. Ion Channels Modulating Gastrointestinal Motility

The neurogenic motility of the GIT is engaged by neurons, Cajal cells, and SMCs. The innervation of the digestive tract consists of the intrinsic ENS and extrinsic nerves from the brain and spinal cord. Neurogenic movements are controlled by neurons, with excitatory and inhibitory neurons forming neural circuits [14]. The intestine is capable of producing peristaltic reflexes and colonic migratory motor complexes in isolation from external neural control. Direct evidence for neuronal control of motility was published. It was possible to control calretinin neurons in the in vivo and ex vivo colon with optogenetic techniques [62] and to achieve the natural elimination of fecal particles. However, control of calretinin neurons in the small intestine did not achieve the desired effect [63], suggesting that the neurons controlling intestinal motility in the small intestine remain unknown. Mechanical forces influence the ENS as shear stress, pressure, and tension [14]. In the mouse intestine, approximately 14% of the neurons in the colon and 22% of neurons in the ileum are mechanosensitive [64]. Like intrinsic primary afferent neurons, mechanosensitive neurons respond to stimuli in various animal models and human samples, indicating that there may be undiscovered pathways for mediating the response to mechanical stimuli. While how mechanosensitive neurons detect stimuli is unclear. However, there is growing evidence that Piezo channels are involved in neuronal mechanoreception [65]. One subpopulation of enteric neurons expresses Piezo2; presumably, the ENS perceives mechanical stimuli directly [66]. More experiments need to be carried out to verify the mechanisms of enteric neurons in mechanotransduction and the control of intestinal motility.

Interest in 5-HT-secreting Piezo2 EECs is gradually increasing, and many pieces of evidence have been found to demonstrate their important role in intestinal motility [67,68]. Single-cell RNA sequencing (ScRNA-seq) results show that Piezo2-positive cells in the intestine are mainly divided into innate immune, lymph endothelial, and EECs [67]. Piezo2-positive EECs can produce 5-HT in the intestinal epithelium and produce contractions in the isolated intestine when optogenetically manipulating Piezo2 EECs. In addition, there is evidence that Piezo2 is sensitive to small intraluminal mechanical stimuli. In vivo experiments, intestinal transit time was increased in Piezo^ΔVil^ and Piezo^ΔTph1^ mice [67,68]. This suggests that Piezo2 acts as an important target for defecation regulation. In the human colon, Piezo2 expression by ECCs decreases with age and mechanical sensitivity [68]. Considering the above study, the diminished intestinal motility may be related to the reduction of Piezo2, and Piezo may serve as a promising drug target to regulate intestinal motility. However, the specific mechanism and therapeutic effects on the disease need further experiments.

Intestinal SMCs have mechanosensory capabilities and receive electrical modulation [69]. Direct activation of SMCs with optogenetics can directly induce contractile activity in the stomach [70]. There is controversy regarding the validity of extracellular recording [71,72]. Isolated cells are not fully representative of the condition in the physiological state, so in vivo techniques are needed. In contrast, transgenics and optogenetics are suitable methods to mark a specific cell type. In vivo electrophysiology is also an excellent technique to maintain the physiological properties of in vivo cells. We should recognize that intestinal SMCs serve as effectors of intestinal motility, and we should recognize their essential role in regulating intestinal motility, and the mechanisms of mechanical stimulation in intestinal SMCs and Cajal cells need to be further elucidated.

The role of 5-HT in the regulation of intestinal motility has been well discussed since the 1960s [73]. After genetically knocking out *Tryptophan Hydroxylase 1 (TPH1)*, 5-HT synthesis is affected and subsequently impeded to transport throughout the intestine [74]. It is a very complex process, from the synthesis of 5-HT to reaching the SMCs to exert its effect. 5-HT is mainly secreted by EECs, a process in which TRPA1 and Piezo2 are involved [17,18]. In addition, some neurons can also secrete 5-HT [75]. And Piezo1 regulates the synthesis of 5-HT by sensing single-stranded RNA [76]. The 5-HT3 receptor (5-HT3R) expressed in the submucosal enteric plexus enables neurons to be triggered by 5-HT and generate electrical activity [77]. Rotavirus-induced alterations in intestinal dynamics depend on 5-HT3R activity, and *5-HT3R*–knockout mice exhibit altered defecation patterns [78]. In embryos, the ENS forms neurite projections to the myenteric plexus and responds to 5-HT signals [79]. ScRNA-seq data corroborate present experiments. 5-HT secretion causes sensory neurons to excite interneurons, which cause motor neurons to release acetylcholine, bradykinin, or nitric oxide (NO) to regulate movement [80].

TRPA1 is expressed in inhibitory motor neurons and regulates motility [81]. Mesenchymal cells in the lamina propria of the colon also express TRPA1, which regulates intestinal motility through the release of PGE2 [82]. Mechanosensitive neurons expressing TRP play an important role in defecation in *Drosophila* [83]. TRPA1 has a multifaceted effect on intestinal motility and does not always stimulate motility [84]. TRPV2 regulates gastric emptying in inhibitory motor neurons [85] and may be related to the mediating role of NO [86]. Optogenetic manipulation of the epithelium and secretion of ATP and 5-HT induces calcium activity in the myenteric plexus and subsequently causes alterations in gut motility [87]. Neurons in the myenteric plexus respond to ATP and 5-HT, and the resulting motor enhancement can be blocked by antagonists of ATP and 5-HT. TRPA1 inhibits gastric emptying [88] but promotes peristalsis and defecation in the colon, and mesenchymal cells in the lamina propria that express TRPA1 may have an important role [82,89,90]. We need to utilize more in vivo methods to evaluate electrophysiological properties rather than just in vitro recording [91]. In addition, the altered gastrointestinal dynamics caused by AITC were questioned as possibly unrelated to TRPA1 [84]. TRPV1 regulates gastrointestinal motility in gastric contraction [92], jejunum motility [93], colonic contraction [94], and afferent nerves [95], and plays a role in motility disorders in IBS [96]. TRPV4 is found in macrophages and epithelium and exerts a regulatory effect on gastrointestinal motility through PGE2, NO, Ca, and ATP [97,98,99].

Maintenance of gastrointestinal motility is essential [100,101]. Neurons play an essential role in defecation [102]. The neural sensation of bowel contents affects the frequency of peristalsis and fecal properties [67]. The potential of ion channels to regulate intestinal dynamics is of great significance for the treatment of dynamics-related diseases.

## 5. Conclusions

**Figure 2:**
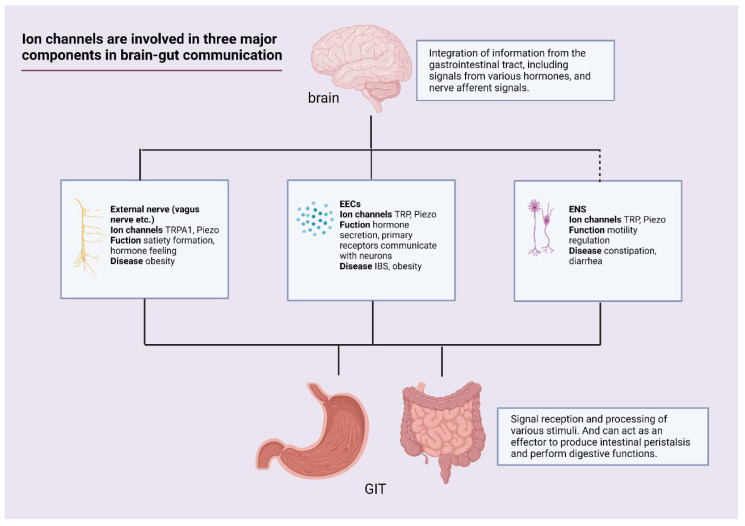
Ion channels involved in three major components in brain-gut communication. There are three important components involved in digestion and feeding regulation, including externally innervated nerves of the gut, EECs, and enteric nervous system (ENS). (1) Externally innervated nerves are able to acquire information in the gut; some neurons can directly sense stimuli, while others acquire information about the state of the gut by sensing hormones secreted by EECs. The regulation of satiety signals can be used to experimentally regulate diseases such as obesity. (2) EECs are important signal receptors in the intestine. They are able to make direct contact with intestinal contents and integrate various signals to regulate their own secretory function. These secreted hormones have important roles in the regulation of intestinal homeostasis. For example, we can use the effect of 5-HT on intestinal dynamics to regulate irritable bowel syndrome (IBS), as well as GLP-1 on obesity. (3) The ENS is an independent part that receives information from EECs and extrinsic nerves and is able to form complex neural loops that control intestinal dynamics and exert a regulatory effect on intestinal dynamics. Further studies on ENS are expected to have good therapeutic targets in controlling intestinal dynamics-related diseases such as constipation and diarrhea. (created with BioRender.com).

Ion channels are important for the homeostasis of the GIT. Ion channels are involved in the secretory activity of EECs, which couple chemical and mechanical stimuli to secretory activity. Nutrients in the intestine provide a large amount of information to the ion channels, which are received by the vagus nerve and the brain to regulate feeding. Ion channels can act as important factors in neurotransmission in brain-gut interaction, and neurons may carry out direct signal sensing through inward currents generated in cells by ion channels. The source of information received by the ENS is inextricably linked to the sensation of ion channels. The discovery of new subpopulations of cells in the intestinal epithelium that sense mechanical and chemical stimuli and their functions are intriguing. Ion channels are an important target of action in gastrointestinal diseases and have good therapeutic potential (Figure 2). Numerous preclinical studies have established the basic framework for the role of mechano- and chemosensitive channels in the GIT. More appropriate ways of regulating ion channels and the utility of regulating ion channels in diseases need further exploration.

## Data Availability

Not applicable.
